# Comparing the signal enhancement of a gadolinium based and an iron-oxide based contrast agent in low-field MRI

**DOI:** 10.1371/journal.pone.0256252

**Published:** 2021-08-17

**Authors:** Jordy K. van Zandwijk, Frank F. J. Simonis, Friso G. Heslinga, Elfi I. S. Hofmeijer, Robert H. Geelkerken, Bennie ten Haken

**Affiliations:** 1 Magnetic Detection & Imaging, TechMed Centre, University of Twente, Enschede, Netherlands; 2 Department of Vascular Surgery, Medisch Spectrum Twente, Enschede, Netherlands; 3 Multimodality Medical Imaging Group, TechMed Centre, University of Twente, Enschede, Netherlands; University of Queensland, AUSTRALIA

## Abstract

Recently, there has been a renewed interest in low-field MRI. Contrast agents (CA) in MRI have magnetic behavior dependent on magnetic field strength. Therefore, the optimal contrast agent for low-field MRI might be different from what is used at higher fields. Ultra-small superparamagnetic iron-oxides (USPIOs), commonly used as negative CA, might also be used for generating positive contrast in low-field MRI. The purpose of this study was to determine whether an USPIO or a gadolinium based contrast agent is more appropriate at low field strengths. Relaxivity values of ferumoxytol (USPIO) and gadoterate (gadolinium based) were used in this research to simulate normalized signal intensity (SI) curves within a concentration range of 0–15 mM. Simulations were experimentally validated on a 0.25T MRI scanner. Simulations and experiments were performed using spin echo (SE), spoiled gradient echo (SGE), and balanced steady-state free precession (bSSFP) sequences. Maximum achievable SIs were assessed for both CAs in a range of concentrations on all sequences. Simulations at 0.25T showed a peak in SIs at low concentrations ferumoxytol versus a wide top at higher concentrations for gadoterate in SE and SGE. Experiments agreed well with the simulations in SE and SGE, but less in the bSSFP sequence due to overestimated relaxivities in simulations. At low magnetic field strengths, ferumoxytol generates similar signal enhancement at lower concentrations than gadoterate.

## Introduction

Contrast agents (CAs) have been used in MRI for decades with a great use for angiographic purposes [1]. Cardiovascular diseases as peripheral arterial disease, aortic aneurysms, and cardiomyopathy benefit from enhanced imaging possibilities due to CA administration with excellent signal-to-noise (SNR) ratios. Gadolinium based contrast agents (GBCAs) are used for generating positive contrast in millions of MRI examinations because of their unique magnetic properties [2]. An important characteristic of CAs in MRI is that their effect depends on the used field strength [3]. At lower magnetic field strengths the molecular tumbling rate is reduced which increases effect of a T_1_ CA [4].

Low-field MRI (0.25-1T) has advantages of lower costs, smaller footprint, and better subject accessibility with respect to higher field strengths [5]. MR on these lower field strengths could also benefit from developments in higher-field MRI over the past decades. The benefits of a high-performance low-field MR system (0.55T) in MRI-guided catheterizations, high susceptibility regions, and efficient image acquisition strategies have been demonstrated [6]. Because of this renewed interest, it has become relevant which CA is best suited for low-field MRI.

The use of CAs in low-field MRI up to now has mainly focused on examinations with gadolinium, which generates positive contrast [3]. This was mainly done in intraoperative MR systems for neurosurgery, which often employ low field strengths [7, 8]. Further applications that may require CA administration at lower field strengths can be found in endovascular interventions [9, 10], enhanced vascular imaging [11], or even in gravity dependent investigations [12]. The lowered field will probably not affect the GBCA behavior [13]. However, not much work has been published on optimizing contrast agents for low-field MRI applications. Besides, when the characteristics of a CA that influence relaxation times stay equal at lower field strengths, the contrast-to-noise ratio will drop because of an inherent lower T_1_ on lower field strengths. This raises the question whether at low field strengths CAs other than GBCAs might be more suitable, such as ultra-small super paramagnetic iron-oxides (USPIOs).

USPIOs possess different physiological and relaxation characteristics compared to GBCAs. When shortening the T_1_, the signal becomes larger (positive contrast) because of faster longitudinal relaxation. Likewise, shortening the T_2_* results in less signal (negative contrast) since the net magnetization decreases faster with shorter T_2_*. Where GBCAs are mostly used for generating positive contrast on T_1_-weighted imaging, USPIOs are frequently used in T_2_* weighted imaging as negative CA because the magnetic susceptibility of their iron core greatly shortens T_2_* [14]. However, when administered at low concentrations, the T_1_ shortening effects of USPIOs dominate the T_2_* shortening effects, leading to a positive contrast. Next to that, the relaxivity of USPIOs (i.e. the amount of change in relaxation rate per concentration) increases greatly and possibly triples at field strengths lower than 1T [15], meaning their effect is larger at lower concentrations. At these lower field strengths their *r*_2_/*r*_1_ ratio is also more favorable to achieve positive contrast [15, 16]. This makes USPIOs an interesting option for a low-field MR system. Besides their improved signal enhancement properties, USPIOs are often given a dextran coating resulting in surface properties that ensure vasculature retention times with half-times up to 21 hours [17] versus 1.5 hours for GBCAs [18]. Next to that, GBCAs are associated with nephrogenic systemic fibrosis and gadolinium accumulation in organs that could be harmful for patients in longer procedures [19, 20]. Ferumoxytol, an USPIO previously used for magnetic resonance angiography has therefore been considered a useful alternative to GBCAs [21–23].

This work combines simulations and experimental testing of an USPIO and a GBCA on low magnetic field strength (0.25T). There have been investigations [13, 24–26] in relaxivities of both contrast agent types on field strengths like 1.5T and 3.0T. An additional signal intensity (SI) gain because of higher relaxivities for the clinically interesting USPIO ferumoxytol [17] on a field strength lower than 1.5T is to be expected. At 0.55T only slightly higher relaxivity was found for ferumoxytol [6], but for ultra-low field MRI (<10mT) USPIOs show more pronounced advantages in terms of enhanced signal and shorter scan times [16, 27]. Forthcoming, at 0.25T the added value of USPIO enhanced imaging should be investigated.

The aim of this study was to investigate whether a gadolinium-based or an iron-oxide based CA is more suited to provide signal enhancement in low-field MRI. For this comparison, SIs were simulated for three different MR sequences that are generally employed in an angiographic or vascular interventional setting. Initial scan parameters were chosen to provide optimal positive contrast in T_1_-weighted images and to avoid saturation. Subsequently, the signal enhancement generated by both CAs was measured in phantom samples on a low-field MRI-scanner and compared to the simulations.

## Materials and methods

The GBCA gadoterate acid (Gd-DOTA, Dotarem®, Guerbet, France) and USPIO ferumoxytol (Feraheme, AMAG Pharmaceuticals, USA) were used as contrast agents in this research. A 0.25T MRI scanner (G-scan Brio, Esaote, Italy) equipped with a coil used for wrist examinations was used in all experiments. For MR angiography, values of 2.67 mM for gadoterate (0.2 mmol/kg) [28] and 0.96 mM of iron (4 mg/kg) [29] are conventional on field strengths equal or higher than 1.5T. Therefore, a phantom both for ferumoxytol and gadoterate was built containing concentrations of 0.15, 0.30, 1.2, 7.0, and 14 mM in bovine blood in a circular setup of 15 ml vials with a fish oil marker to indicate the orientation of the samples. A sample of bovine blood without CA served as reference. First, the relaxivities of both CA’s on 0.25T were experimentally estimated. Secondly, their SIs were simulated for common MR sequences. Lastly, phantom experiments were performed on the 0.25T MRI scanner to validate the simulated signal intensities.

### Relaxivity

Since the *r*_*1*_ and *r*_*2*_ values of both ferumoxytol and gadoterate were unknown on 0.25T, this was measured on a 0.25T MRI scanner using the concentrations range of CAs and the NOVIFAST method [30] for *r*_*1*_ and DESPOT2 [31] for *r*_*2*_. Scanning parameters for these methods are given in Table 1. First, with NOVIFAST we used a spoiled gradient echo sequence (SGE) with six varying flip angles between 10° and 90° to obtain T_1_ maps of the samples. Subsequently, the *r*_*1*_ values were calculated using Eq 1.


$$\frac{1}{T_{i}(C)}=\frac{1}{T_{i}(0)}+r_{i}*C\;\mathrm{with}\;i=1,2$$ [1]

**Table 1:** Parameters of the NOVIFAST and DESPOT2 methods that were applied to obtain consecutively T_1_ and T_2_ maps.

	Sequence	TR (ms)	TE (ms)	FA (°)
**NOVIFAST**	SGE	26	15	10-20-30-40-60-90
**DESPOT2**	bSSFP	10	5	20-30-40-50-70-90

For calculation of the *r*_*2*_ values with the DESPOT2 method the T_1_ values resulting from NOVIFAST were required as input. We obtained balanced steady-state free precession (bSSFP) scans with varying flip angles ranging from 20° to 90°. The resulting T_2_ maps were used for determination of the *r*_*2*_ values using Eq 1.

The *r*_*1*_ and *r*_*2*_ values of ferumoxytol on 1.5T are known from literature (*r*_*1*_ = 15 mM^-1^ s^-1^ and *r*_*2*_ = 89 mM^-1^ s^-1^) [32]. Relaxivity for gadoterate on 1.5T has been described by Rohrer [13] as *r*_*1*_ = 2.9 mM^-1^ s^-1^ and *r*_*2*_ = 3.2 mM-1 s-1.

### Sequences & simulations

SIs were simulated for three relevant sequences to study the SI as a function of the CA concentration. Simulations were performed at low field (0.25T) and at common field strength (1.5T) for comparison.

First, a regular spin echo (SE) that is often used for anatomical reference was simulated. Eq 2 states the SI in a SE in relation to the proton density (PD), repetition time (TR), echo time (TE), and T_1_ and T_2_ values of the tissue [33]. To obtain maximum T_1_-weighted contrast for optimal contrast agent visibility, TR and TE were set to the lowest possible values of the MRI scanner (Table 2). Furthermore, a flip angle of 90° was chosen.


$${SI}_{SE}(C)=PD*(1-e^{-TR/T_{1}(C)})*e^{-TE/T_{2}(C)}$$ [2]

**Table 2:** Parameters of the MR sequences that were used to analyze the samples with different concentrations of ferumoxytol and gadoterate. Acq. res. = acquired resolution.

	SE	SGE	bSSFP
**TR (ms)**	50	26	10
**TE (ms)**	18	10	5
**FA (°)**	90	40	60
**Num. acquisitions**	1	1	3
**Slice thickness (mm)**	10	10	10
**Acq. res. (mm x mm)**	0.78×0.78	0.78×0.78	0.98×0.98

Second, an SGE sequence that can be used for angiographic purposes was simulated. Its signal is defined by Eq 3 [34].


$${SI}_{SGE}(C)=PD*\frac{\sin\theta (1-e^{-TR/T_{1}(C)})}{1-\cos\theta e^{-TR/T_{1}(C)}}e^{-TE/{T_{2}}^{*}(C)}$$ [3]

The measured signal in this sequence is also dependent on the flip angle (θ). To avoid saturation of the sample with large flip angle, we used a flip angle of 40° in the simulations and experiments.

Third, a bSSFP was simulated because of its favorable SNR characteristics. Less contrast difference due to CAs is expected for this sequence since its contrast is known to be T_2_/T_1_-weighted. Eq 4 gives the SI expression for bSSFP [35].


$${SI}_{SSFP}(C)=PD*\sin\theta \frac{1-e^{-TR/T_{1}(C)}}{1-(e^{-TR/T_{1}(C)}-e^{-TR/T_{2}(C)})\cos\theta -(e^{-TR/T_{1}(C)})(e^{-TR/T_{2}(C)})}e^{-TE/T_{2}(C)}$$ [4]

Signal of all sequences was normalized with respect to the situation where no CA was added (C = 0) to accentuate the effect of the CA. The main outcome is the ratio of increase in SI with respect to SI(0). Table 2 shows the used scan parameters for all sequences. All simulations were validated by scanning both ferumoxytol and gadoterate phantoms.

### Analysis

The data were analyzed with Matlab (Mathworks Inc., Natick, USA). Signal intensities were measured from the average of automatically selected regions of interest with a radius of 6 pixels around the center of each sample to avoid signal affected by Gibbs ringing. Subsequently, the values were normalized with respect to the intensity of the reference sample. The coefficient of variation of the signal intensity in the samples was obtained by dividing the SD by the mean SI in each sample.

## Results

**Fig 1:**
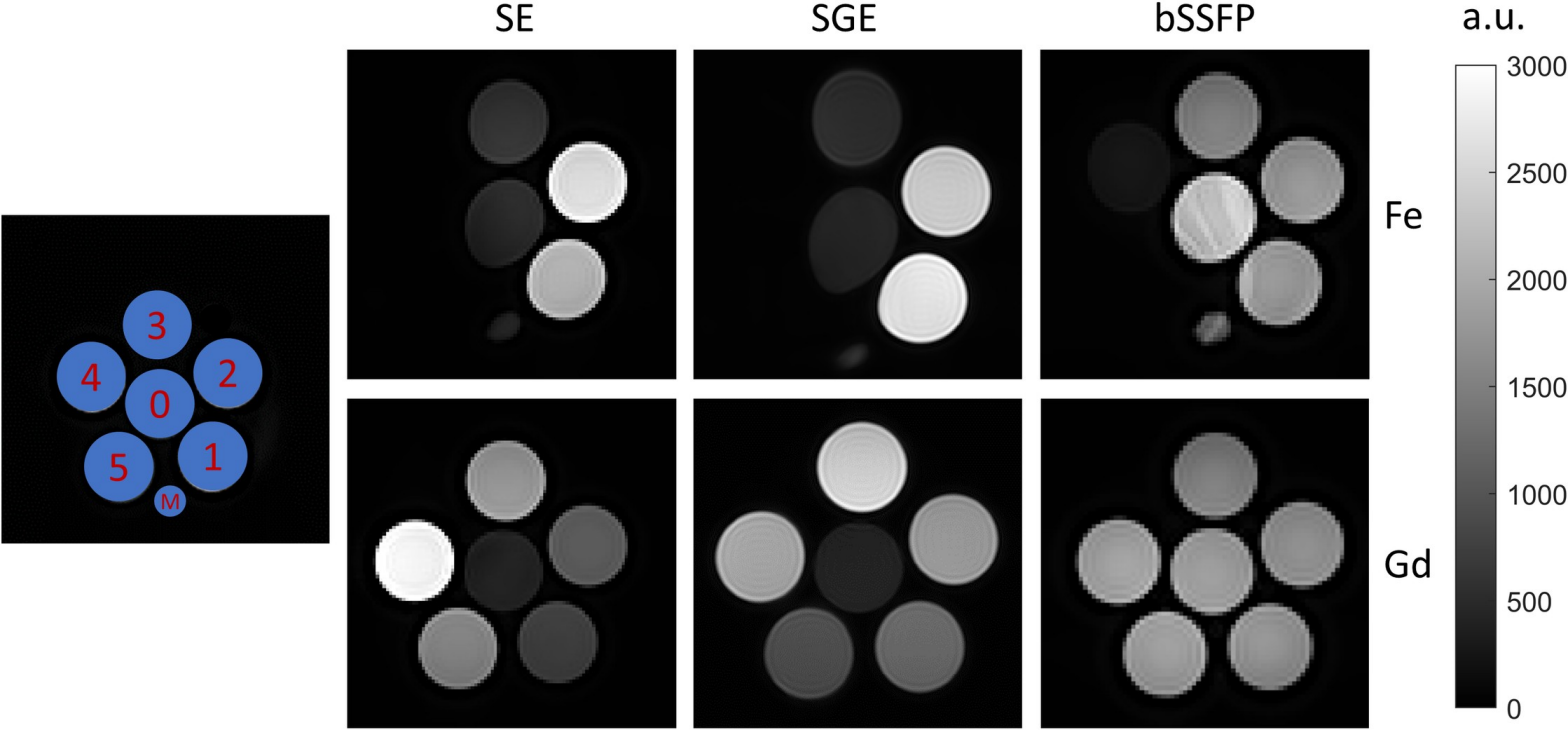
**Setup of the CA phantom (left) for both ferumoxytol (upper row; Fe) and gadoterate (lower row; Gd), with 0 = reference (no CA), 1 = 0.15 mM, 2 = 0.3 mM, 3 = 1.2 mM, 4 = 7 mM, 5 = 14 mM.** ‘M’ indicates the marker for orientation. MRI scans of the SE (middle left), SGE (middle right), and bSSFP (right) sequence show the amount of signal compared to the reference sample which is in the center of the setup. Deformation of the reference sample can be seen in the SE and SGE scans of ferumoxytol. Due to scaling only images made with the same sequence can be compared with respect to signal intensity.

Fig 1 shows the MRI scans of ferumoxytol and gadoterate samples for the three sequences. The coefficient of variation in the samples was 1.3% (SE), 0.9% (SGE), and 1.1% (bSSFP). Noticeable is the susceptibility artefact around the samples of 7 and 14 mM ferumoxytol, which partly distorted the reference sample (see Fig 1, top row). Moreover, the magnetic field inhomogeneities due to the highly paramagnetic samples cause banding artefacts to appear in the bSSFP scans of the ferumoxytol samples. There was no signal in the samples with high concentration (7 mM and 14 mM) of ferumoxytol.

**Fig 2:**
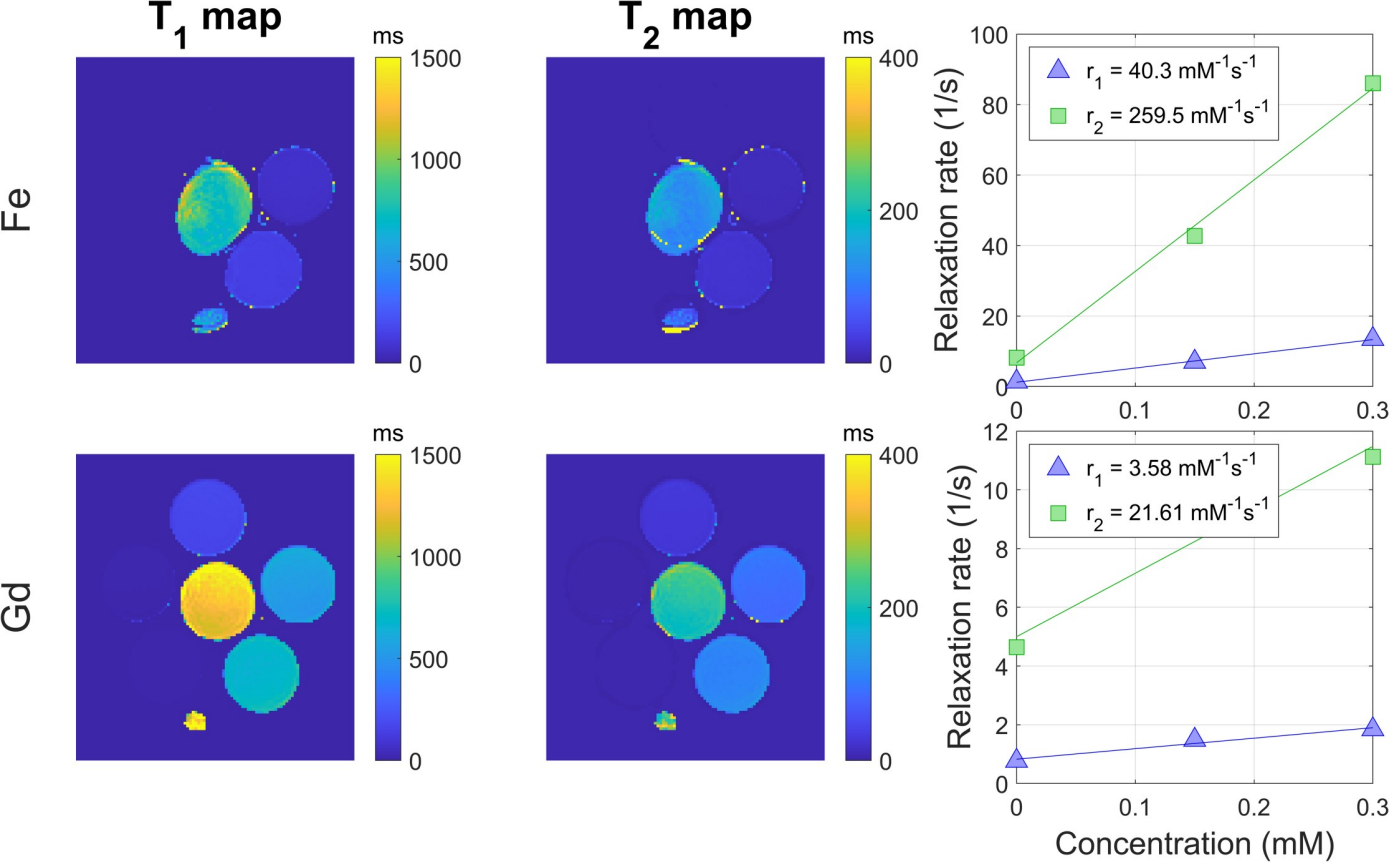
**T**_**1**_**maps (left column) that were calculated with NOVIFAST and used as input for the DESPOT2 method to generate the T**_**2**_**maps (middle column).** T_1_ and T_2_ values in the samples were used to calculate relaxation rates as function of the concentration (right column). For ferumoxytol (top row) and gadoterate (bottom row) relaxivity values were fit based on the relaxation rates.

The T_1_ and T_2_ maps together with the fit relaxivity values are shown in Fig 2. For ferumoxytol we found an *r*_*1*_ of 40.3 mM^-1^ s^-1^ and an *r*_*2*_ of 259.5 mM^-1^ s^-1^ at 0.25T (both with R-squared = 0.99). For gadoterate, we found an *r*_*1*_ of 3.58 mM^-1^ s^-1^ and an *r*_*2*_ of 21.6 mM^-1^ s^-1^ at 0.25T (both with R-squared = 0.96). These values were used as input for simulations of SI curves for SE, SGE, and bSSFP.

**Fig 3:**
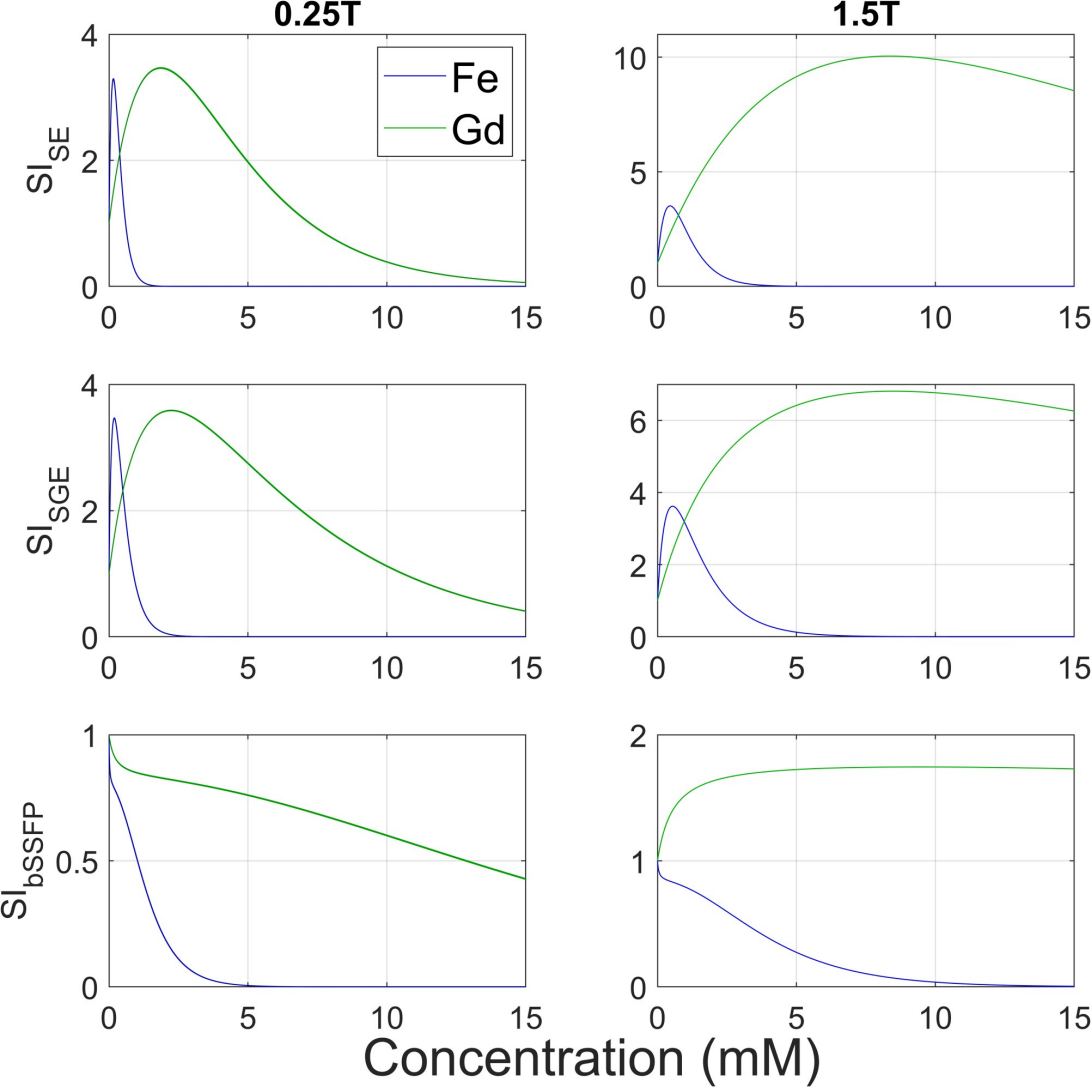
**Simulated SIs calculated from 0–15 mM for ferumoxytol (Fe) and gadoterate (Gd) for an SE, SGE, and bSSFP sequence at 0.25T (left column) and 1.5T (right column).** SIs are shown as normalized values as ratio to no CA (which corresponds with a value of 1). These simulations contributed to identifying relevant concentrations (0–0.15–0.30–1.20–7.0–14.0 mM) that were prepared to perform the experiments. Note the difference in y-axis between 0.25T and 1.5T and the lack of signal enhancement in the bSSFP sequence on 0.25T.

Fig 3 shows the simulated SI curves for different concentrations of ferumoxytol and gadoterate on 0.25T and 1.5T. The curves of SE and SGE have similar shapes for ferumoxytol with a sharp peak of signal enhancement at low concentrations. For gadoterate there is a plateau at higher concentrations indicating a broader range of concentrations that gives similar signal enhancement on both field strengths. The bSSFP simulation shows for both CAs a decrease in signal compared to the reference, meaning that no signal enhancement could be achieved. A concentration of around 0.16 mM ferumoxytol yielded the maximum signal increase of 3.3 times the reference at 0.25T, whereas for gadoterate this was 3.5 times at a concentration of 1.9 mM on SE and SGE on 0.25T. Maximum increases are thus comparable for both CAs, but occurred earlier for ferumoxytol. The amount of potential maximal signal increase was similar on 0.25T and 1.5T for ferumoxytol, but higher for gadoterate on 1.5T with a 10-fold (SE) and 6.8-fold (SGE) increase.

**Fig 4:**
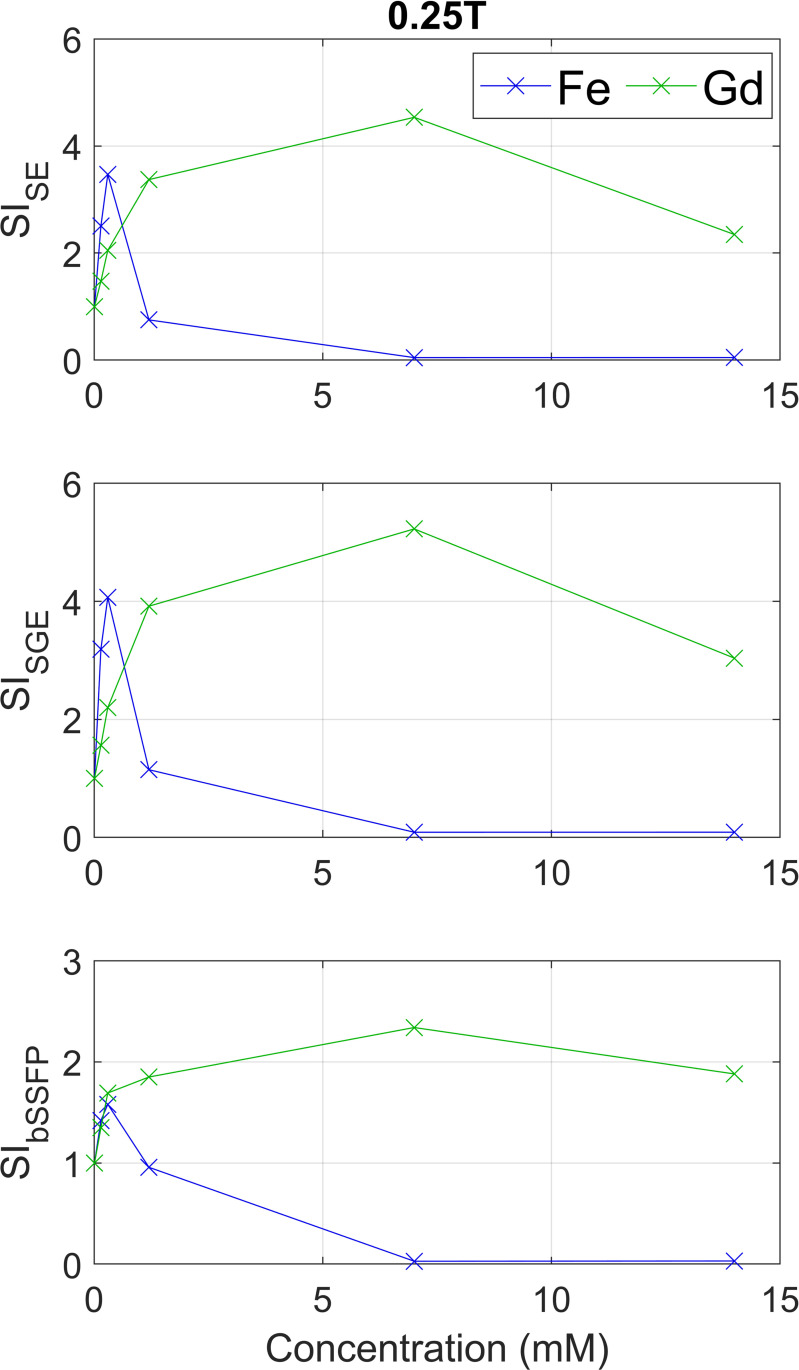
Measured signals (SIs) for the prepared concentrations (0–0.15–0.30–1.20–7.0–14.0 mM) for an SE, SGE, and bSSFP sequence at 0.25T. SIs are shown as normalized values as ratio to no CA (which corresponds with a value of 1). These results were compared with the normalized simulated SIs from Fig *3* (left column).

Fig 4 shows the measured SIs for both CAs on all three sequences on 0.25T. Visual comparison with the simulated curves shows similar results with a peak in SI at low concentrations of ferumoxytol and a wider peak at higher concentrations for gadoterate on SE and SGE. The measured bSSFP shows for ferumoxytol a maximum increase of 1.6 times the reference in the 0.30 mM sample, whereas all gadoterate samples show increase with a maximum of 2.3 times for the 7 mM sample. This is in contrast with simulations which predicted no enhancement at all.

## Discussion

The goal of this research was to measure whether a GBCA (gadoterate) or an USPIO (ferumoxytol) would be more suitable for providing positive contrast at low magnetic field strengths. This research confirmed that a low concentration of ferumoxytol (<0.40 mM) leads to similar signal enhancement as much higher concentrations of gadoterate (around 5 mM) on these field strengths.

Relaxivities at 0.25T were measured based on T_1_ and T_2_ maps that were calculated with the NOVIFAST [30] and DESPOT2 [31] methods respectively. Both methods seemed to be accurate based on the R-squared values when fitting through the different samples. The T_1_ maps seemed to produce *r*_*1*_ results in line with expected relaxivities after extrapolation of 0.5T and 1.5T data [6, 13]. However, the calculated *r*_*2*_ was much higher than expected. Based on known relaxivities on 0.5T and 1.5T, these *r*_*2*_ of 259.5 mM^-1^ s^-1^ (ferumoxytol) and 21.6 mM^-1^ s^-1^ (gadoterate) were expected to be in the range of 80–120 mM^-1^ s^-1^ and 3–5 mM^-1^ s^-1^ respectively [6, 13].We hypothesize that this is due to signal loss in bSSFP; when this sequence is incorrectly balanced due to background gradients and a relatively long TR, the signal can become T_2_* (instead of T_2_) dependent [36]. This results in a severe underestimation of T_2_ leading to an overestimated *r*_*2*_. Since the simulations were based on these overestimated *r*_*2*_ values, simulated signal in the bSSFP sequence is lower than the measured signal. This was seen and confirmed in the experiments, where a slight increase in SI for both CAs was observed. When simulations were performed with the *r*_*2*_ values expected from literature, simulated signals were more like the experiments.

The relaxation effects that were investigated in this study are harnessed differently for an SE, SGE and bSSFP sequence. An important factor when selecting and applying a CA to specific environments that should be taken into account is the weighting of the sequence [4, 37]. For angiographic purposes, often T_1_-weighted sequences are used like the SE and SGE. Contrast of those sequences can be enhanced by using a CA with high *r*_*1*_ to strongly influence signal intensity in the blood. However, for bSSFP sequences that are often used in abdominal and cardiac imaging, the contrast depends on the T_2_/T_1_ ratio. Administration of CAs is then only beneficial when the T_2_/T_1_ ratio does not approach unity [37].

The chosen imaging parameters were determined by the lowest possible combination of TR/TE on the used MRI system. Although these parameters could be further minimized on other MR systems, the experiments demonstrated higher SI at low concentrations of ferumoxytol (<1.2 mM) than at higher concentrations (> 7.0 mM) of gadoterate.

Although ferumoxytol seems to be advantageous at low field strengths when used in low concentrations, it can have certain drawbacks. For example, in dynamic contrast enhanced MRI the application uptake curve is fit to characterize certain tissue properties, and it is often required to run more than one CA dose [38]. To this aim, a faster CA washout is required. Since USPIOs show considerably slower washout times, it may be less convenient for such applications. Furthermore, the narrow concentration range at which the SI peaks has the potential drawback that the concentration should not be too high after administration since this will weaken SI. However, careful administration of USPIO doses is in line with its safety regulations, meaning that high doses are already not allowed. Besides, ferumoxytol blood-pool residence time in comparison with GBCAs is much longer, which negates the need for administration of subsequent doses [29].

The observed narrow peak in Fig 3 in the simulations for ferumoxytol in SE and SGE implicates that only low concentrations cause signal increase, whereas higher concentrations would lead to signal decrease. Besides, the measurements may be missing the absolute maximum of the peak because sampling points are scarce. The optimal concentration range for gadoterate is achieved at higher concentrations under a wider range due to moderate *r*_*2*_ relaxivity. Literature supports our findings by stating that ferumoxytol is an interesting choice as intravascular contrast agent at lower field strengths [6].

Clinical examples that could benefit from lowered CA administration are MR interventions, vascular imaging, and situations where GBCAs are not desired or even impossible to use because of kidney disease. Besides, the difference in CA excretion mechanism between ferumoxytol and GBCAs can also be exploited [39]. Whereas the half-life of ferumoxytol is more than 15 hours, gadolinium CAs have half-lives of around 70–120 minutes [40]. This longer blood circulation time of ferumoxytol will be an advantage in a situation where a constant SI increase is required over a longer time, like in an endovascular intervention with a length of hours [41]. A high peak in SI for low concentrations is then even more useful, since it means that less CA has to be administered over time, decreasing toxicity. Furthermore, lower doses of ferumoxytol at lower field strengths have clinical benefits in terms of reduced adverse reactions, and less hepatic uptake giving less confounding signal changes in other MRI scans of the patient.

The additional benefit of the long blood circulation time of ferumoxytol is that it enables the user to exploit longer imaging times. This allows for more averaging during acquisition resulting in higher SNR, which is also desirable when scanning at low field strengths. Further research could address SNR optimization in low-field MRI to facilitate clinical application of USPIO enhanced imaging.

## Conclusions

In conclusion, solely based on its relaxivity characteristics ferumoxytol is more beneficial in generating positive contrast at low magnetic field strengths than gadoterate where lower concentrations yield almost equal signal enhancement. MR sequence optimalisation with respect to specific USPIO behavior in vivo addressing both excretion mechanism and retention time should be the subject of subsequent research.

## Supporting information

S1 Dataset (ZIP)
