# Supplementary material for: Comparing the signal enhancement of a gadolinium based and an iron-oxide based contrast agent in low-field MRI
Source: PLoS One. 2021 Aug 17;16(8):e0256252. doi: 10.1371/journal.pone.0256252 (PMC8370648; doi:10.1371/journal.pone.0256252)
Supplement: Attachment — Submitted filename: Response to Reviewers.docx [file pone.0256252.s002.docx]

**Answers on both reviewers comments were numbered and in the manuscript for each change it was indicated to which comment the change corresponds. With the text in bold our replies are stated below.**

Reviewer #1:

This is a technically sound and clearly documented investigation into the relaxivity of ferumoxytol and gadoterate at 0.25 T in phantoms accompanied by modelling to explain how the experimental results map onto a broader range of conditions.

The basis of the work is not very original, and the paper could be improved with more rigorous academic referencing of past work to position these results in context, but the results at 0.25 T seem to fill a small gap in the literature and will be of some benefit to an audience interested in applying similar contrast agents in this low magnetic field regime.

**Many thanks for your efforts in thoroughly reviewing this paper and your kind words. I have tried to answer your questions and remarks below and have adjusted the corresponding text in the manuscript.**

In terms of how the authors could generally improve their paper, I would recommend they expand their discussion of relaxation mechanisms at low field and how these relaxation effects are harnessed by particular imaging sequences. I would recommend the review article by Wahsner et al. (<https://doi.org/10.1021/acs.chemrev.8b00363>) as a good starting point for the broader context of selecting and applying contrast agents to specific environments.

**0.1 Thanks for pointing out this interesting review. We expanded our discussion (as also indicated by the responses to specific criticisms below). Relaxation effects and their relation to MR imaging sequences were handled more extensively.**

I have few specific criticisms of the prose of the paper as a whole, which is largely written clearly and concisely, but I would like to mention a few specific issues that occurred to me:
1. On line 89 the authors say " However, an additional signal intensity (SI) gain because of higher relaxivities for the clinically interesting USPIO ferumoxytol [16] on a field strength lower than 1.5T has not been investigated." I find it a little unclear what the authors are trying to say here but I know for certain that USPIOs have been investigated at fields lower than 1.5T. Two investigations that spring to mind, and are particularly relevant to many of the points this paper makes are Yin et al. (<https://doi.org/10.1038/s41598-018-30264-5>) and Waddington et al. (DOI: 10.1126/sciadv.abb0998).

**1.1 We realize that there have been some investigations on lower field strengths and we should point this out. The proposed studies are performed at ultra-low field (<10mT), since in our study we make use of a 0.25T field strength, this is orders of magnitude different. However, those studies are very much relevant and are therefore mentioned now with respect to the current study.**

2. The paragraph beginning at line 217 seems unclear to me and I think would benefit from more explanation of how the bSFFP signal intensity varies with sequence parameters and the relative relaxivities of the samples. I think at several other points the rest of the paper would also benefit from this explanation, as that would help explain the banding artifacts and distortion seen in Fig 1.

**1.2 The paragraph you mentioned seems indeed a bit unclear at first. Our reasoning why the bSSFP signal that we measured did not match with the simulations was that other factors like field inhomogeneities influence the signal more in bSSFP sequences. This also causes the banding that was sharply noticed as visible in Fig 1. We now elaborated more on why the experimental bSSFP signal was contradicting with the simulations. This explanation guides the reader further in understanding for what sequences USPIOs would be beneficial and what the drawbacks are of applying a bSSFP while using USPIOs.**

3. The figures generally lack explanatory labels and helpful annotations to aid a reader and in the case of Fig1 there are also no colourbars. I appreciate that comparing across images may not always be a useful or fair comparison but I believe that it is crucial to still show the colour scales for each image so a reader can better interpret and evaluate the quality of the image.

**1.3 Because all images were equally scaled and the units are arbitrary we first decided to not include a colorbar. However, to make clear that this is the case, we agree interpretation of the images would indeed benefit from this and we added the colorbar. Furthermore, Fig 2 and its caption were revised with removal of abundant information and (white) space. This was also in line with the comments of reviewer #2 on the lack of a colorbar and lower image quality. Fig 3 and Fig 4 were exported in higher quality and their captions were supplemented where necessary. Besides, the width of Fig 4 is now more compressed in order to compare better with the results in Fig 3.**

Reviewer #2: This paper evaluates the effects on signal intensity due to a popular MRI contrast (agent gadoterate acid) and a promising alternative solution (ultra-small superparamagnetic iron-oxides) when images are taken in a low-field (0.25 T) MRI system. The research here undertaken is important to improve the performance and patient safety of low-field MRI systems when exploiting the benefits of contrast agents. The study compares simulations with phantom measurements for three MRI sequences often used with contrast agents. Results indicate that ultra-small superparamagnetic iron-oxide contrast agents can induce similar positive signal enhancement than as the gadolinium based counterpart, but with less contrast agent concentration, which can be beneficial for certain clinical imaging needs. The manuscript reads easily and the methods employed are appropriate.

**Many thanks for your kind words and efforts in thoroughly reading our manuscript. The issues that you raised were well considered and below we explained and discussed how we improved our manuscript based on your advice.**

Mayor issues:
p. 11, line 224 - From the SI vs CA plot of figures 3 and 4, it is possible to see that having the right concentration is crucial for USPIO because the SI gain is achieved over a narrower concentration range. Although simulations and measurements show qualitatively similar relaxivity curves, the CA concentration needed on measurements is 3 times larger than on simulations. Authors comment that this is likely due to an underestimation of T2 values. This argument would be solid if it would be estimated what is the T2 underestimation ratio that would justify the required concentration difference and if this is matches with what can be achieved by their MRI system.

**2.1 This is an excellent issue that you are pointing out. First of all, the width of Fig 3 and Fig 4 was not matching, making it harder to interpret and compare the maximum SI as function of concentration between experiments and simulations. Where Fig 4 has only three plots with CA ranging from 0-15 mM, Fig 3 has six plots and thus more compressed graphs. We fixed Fig 4 so it matched the range in Fig 3 better.**

**The text regarding the underestimation of T_2_ values in the discussion (original starting on line 224) was also adjusted based on the comments of Reviewer #1. We elaborated on why this underestimation occurred and what it meant for our data. Since magnetic field imperfections led to lower signal values in the bSSFP sequence that were used for calculation of the T_2_ values, the estimated *r_2_* relaxivity was probably much higher than the true value. We explained in this paragraph that the actual *r_2_* of ferumoxytol and gadoterate are more likely between 80-120 and 3-5 mM^-1^ s^-1^ respectively. Simulations using these extrapolated literature values does result in curves that are much more like the experimental curves. The exact T_2_ values that we found for ferumoxytol and that were used in the fit are 123, 23, and 11 ms. T_2_ values of 123, 52, and 26 ms would result in an expected relaxivity of 100 mM^-1^ s^-1^. For such short T_2_ values a small difference causes rapid offset in the *r_2_* fit. However, the signal we measured was more T_2_^*^ dependent and thus smaller than T_2_, as we explained in the discussion. This offset seemed logical in our view, but based on your issue we made our explanation regarding this much more clear.**

p. 12, line 267- Authors conclude that ‘the use of ferumoxytol is more beneficial in generating positive contrast at low magnetic field strengths than gadoterate because lower concentrations yield equal signal enhancement’. This affirmation is misleading as it only considers a concentration advantage of USPIO, but does not account for other arguments in favor and against this contrast agent.

**2.2 Our conclusion was indeed somewhat misleading as we considered the advantage based on relaxivity characteristics alone. We made this more specific in our conclusion.**

Minor issues:
p2, line 39- In the abstract it is not mentioned that, besides lower absolute SI increases, the measurements also show considerably lower relaxivities.

**2.3 You are correct in pointing out that the results section in our abstract did not fully match the results from the manuscript. The estimated *r_2_* relaxivity was indeed very high (probably due to imperfections in our system), which was reflected in the simulations. However, in the final measurements the signal of course depends on the real relaxivity of the CAs. These lower relaxivities in the experiments are causing a mismatch, which was mainly visible in the bSSFP graphs. We corrected this sentence in the abstract.**

p3, line 67- Which characteristics 'stay equal'? Their effect on relaxation times?

**2.4 Yes their effect on relaxation times. We made this more specific in the manuscript.**

p4, line 73- It would help the reader to briefly remind the reader why a reduction in T1 time generates positive contrast and a reduction in T2* generates negative contrast.

**2.5 This is a very nice point to address and greatly helps our reader in understanding the study. We added some sentences on that location to make this more clear.**

p4, line 85- What does MRA stand for?

**2.6 Magnetic resonance angiography. We removed the acronym since it was only used once.**

p4, line 89- Typo ‘contrast agents types’

**2.7 Fixed.**

p7, line 137- Please, fix the missing reference.

**2.8 Fixed. (was due to converting by the upload system)**

p8, line 155- The table should go after its mention on the text, not before.

**2.9 The missing reference was also Table 2, so this mention is now correct.**

Discussion - It would enrich the discussion adding disadvantages of USPIO. For example, a very important application of CBCA is dynamic contrast enhanced (DCE) MRI. On this application the uptake curve is fitted to characterise certain tissue properties, and it is often required to run more than one CA dose. To this aim, a faster CA washout is beneficial. Showing USPIO a considerably slower washout time, it may be less convenient for such applications.

**2.10 We agree this point is valuable to add to the discussion, so we added a paragraph where this relevant drawback of the longer retention time was mentioned.**

Discussion: It would be interesting to discuss whether the narrower concentration range at which the SI peaks has advantages and/or drawbacks. How important is the concentration on diagnosis with these contrast agents? Could some parts of the image have reduced SI enhancement due to too high/low concentration?

**2.11 There are indeed some more drawbacks and advantages related to using the right concentration in clinical practice. For example, a narrow peak means that the concentration should be in that range and in that range only to give positive contrast. Although this seems hard to achieve, the blood-pool residence time of ferumoxytol is much longer compared to GBCAs, making this easier to achieve. Subsequent doses (at least on the same day of imaging) are also not necessary. After administration of well-known doses, ferumoxytol spreads evenly in the blood-pool. We also referred now to the mini-review of Vasanawala et al. (2016, MRM), which elaborates further on safety and techniques of ferumoxytol administration.**

p10, line 215- Typo ‘as’ → ‘at’.

**2.12 We apologize for the confusion. We meant to say that a low concentration of ferumoxytol leads to similar signal enhancement as a much higher concentrations of gadoterate. Nevertheless, this sentence has been made a bit shorter and hopefully more clear.**

p11, line 237- Measurements may be missing the maximum of the peak on the USPIO phantom because sampling points are scarce.

**2.13 That is correct, and we clarified this in the discussion.**

p11, line 240- Please, rephrase.

**2.14 Rephrased the sentence.**

p12, line 267- As mentioned before, using USPIO has benefits but can also have downsides (e.g, multi-dose DCE MRI). It may be convenient to rephrase the sentence not to mislead the reader. Also, the results do not show 'equal' signal enhancement as estated. The USPIO measurements reaches <%25 less signal enhancement than those of GD. See maximum values on figure 4.

**2.15 We edited the text in the conclusion to match this comment and the major issue that was mentioned earlier.**

Fig. 2- Although having a fixed colorbar range often helps comparing the different maps, in this case the range of the T2 maps is much smaller than that of the T1 maps. Visualisation of the T2 maps could benefit from adjusting the colorbar to a shorter range on both Gd and Fe T2 maps. Also, the text on these plots is unreadable due to image resolution. It may have been degraded by the submission system. Please, double check.

**2.16 Fig 2 was extended with a color bar with ranges corresponding to the values that were found in both maps. Also, the caption was clarified which was in line with the remarks of Reviewer #1. All figures were double checked on export in highest possible quality.**

**Because of the thorough review of our manuscript we came across the following paragraph (original starting at p. 11 line 243) in our discussion that has become obsolete. This information was based on previous experiments that were conducted for this manuscript in a demi-water solvent. However, with bovine blood the absolute difference between simulations and experiments was less pronounced. We apologize for this insufficiency, and have removed those sentences.**
