# Supplementary material for: Comparing the signal enhancement of a gadolinium based and an iron-oxide based contrast agent in low-field MRI
Source: PLoS One. 2021 Aug 17;16(8):e0256252. doi: 10.1371/journal.pone.0256252 (PMC8370648; doi:10.1371/journal.pone.0256252)
Supplement: Peer review history

# Peer review: Decision Letter 0

**DOI:** 10.1371/journal.pone.0256252.r001

**Authors:** Viktor Vegh

16 Jun 2021

PONE-D-21-15569

Comparing the Signal Enhancement of a Gadolinium Based and an Iron-Oxide Based Contrast Agent in Low-Field MRI

PLOS ONE

Dear Dr. van Zandwijk,

Thank you for submitting your manuscript to PLOS ONE. After careful consideration, we feel that it has merit but does not fully meet PLOS ONE’s publication criteria as it currently stands. Therefore, we invite you to submit a revised version of the manuscript that addresses the points raised during the review process.

==============================

ACADEMIC EDITOR: Your paper was well received by two reviewers, both making recommendations (one minor and one major) to improve clarity and interpretation of results. Please address all the major and minor points raised by the two reviewers. I've deemed the corrections addressable within weeks, as such my overall recommended is a minor revision.

==============================

Please submit your revised manuscript by Jul 31 2021 11:59PM. If you will need more time than this to complete your revisions, please reply to this message or contact the journal office at plosone@plos.org. When you're ready to submit your revision, log on to https://www.editorialmanager.com/pone/ and select the 'Submissions Needing Revision' folder to locate your manuscript file.

Please include the following items when submitting your revised manuscript:

- A rebuttal letter that responds to each point raised by the academic editor and reviewer(s). You should upload this letter as a separate file labeled 'Response to Reviewers'.
- A marked-up copy of your manuscript that highlights changes made to the original version. You should upload this as a separate file labeled 'Revised Manuscript with Track Changes'.
- An unmarked version of your revised paper without tracked changes. You should upload this as a separate file labeled 'Manuscript'.

If you would like to make changes to your financial disclosure, please include your updated statement in your cover letter. Guidelines for resubmitting your figure files are available below the reviewer comments at the end of this letter.

If applicable, we recommend that you deposit your laboratory protocols in protocols.io to enhance the reproducibility of your results. Protocols.io assigns your protocol its own identifier (DOI) so that it can be cited independently in the future. For instructions see: http://journals.plos.org/plosone/s/submission-guidelines#loc-laboratory-protocols. Additionally, PLOS ONE offers an option for publishing peer-reviewed Lab Protocol articles, which describe protocols hosted on protocols.io. Read more information on sharing protocols at https://plos.org/protocols?utm_medium=editorial-email&utm_source=authorletters&utm_campaign=protocols.

We look forward to receiving your revised manuscript.

Kind regards,

Viktor Vegh, Ph.D.

Academic Editor

PLOS ONE

Journal Requirements:

When submitting your revision, we need you to address these additional requirements.

1. Please ensure that your manuscript meets PLOS ONE's style requirements, including those for file naming. The PLOS ONE style templates can be found at

https://journals.plos.org/plosone/s/file?id=wjVg/PLOSOne_formatting_sample_main_body.pdf and

https://journals.plos.org/plosone/s/file?id=ba62/PLOSOne_formatting_sample_title_authors_affiliations.pdf

2. Please review your reference list to ensure that it is complete and correct. If you have cited papers that have been retracted, please include the rationale for doing so in the manuscript text, or remove these references and replace them with relevant current references. Any changes to the reference list should be mentioned in the rebuttal letter that accompanies your revised manuscript. If you need to cite a retracted article, indicate the article’s retracted status in the References list and also include a citation and full reference for the retraction notice.

3.  In your Data Availability statement, you have not specified where the minimal data set underlying the results described in your manuscript can be found. PLOS defines a study's minimal data set as the underlying data used to reach the conclusions drawn in the manuscript and any additional data required to replicate the reported study findings in their entirety. All PLOS journals require that the minimal data set be made fully available. For more information about our data policy, please see http://journals.plos.org/plosone/s/data-availability.

Upon re-submitting your revised manuscript, please upload your study’s minimal underlying data set as either Supporting Information files or to a stable, public repository and include the relevant URLs, DOIs, or accession numbers within your revised cover letter. For a list of acceptable repositories, please see http://journals.plos.org/plosone/s/data-availability#loc-recommended-repositories. Any potentially identifying patient information must be fully anonymized.

Important: If there are ethical or legal restrictions to sharing your data publicly, please explain these restrictions in detail. Please see our guidelines for more information on what we consider unacceptable restrictions to publicly sharing data: http://journals.plos.org/plosone/s/data-availability#loc-unacceptable-data-access-restrictions. Note that it is not acceptable for the authors to be the sole named individuals responsible for ensuring data access.

We will update your Data Availability statement to reflect the information you provide in your cover letter.

[Note: HTML markup is below. Please do not edit.]

Reviewers' comments:

Reviewer's Responses to Questions

**Comments to the Author**

1. Is the manuscript technically sound, and do the data support the conclusions?

The manuscript must describe a technically sound piece of scientific research with data that supports the conclusions. Experiments must have been conducted rigorously, with appropriate controls, replication, and sample sizes. The conclusions must be drawn appropriately based on the data presented. 

Reviewer #1: Yes

Reviewer #2: Yes

**********

2. Has the statistical analysis been performed appropriately and rigorously? 

Reviewer #1: Yes

Reviewer #2: Yes

**********

3. Have the authors made all data underlying the findings in their manuscript fully available?

The PLOS Data policy requires authors to make all data underlying the findings described in their manuscript fully available without restriction, with rare exception (please refer to the Data Availability Statement in the manuscript PDF file). The data should be provided as part of the manuscript or its supporting information, or deposited to a public repository. For example, in addition to summary statistics, the data points behind means, medians and variance measures should be available. If there are restrictions on publicly sharing data—e.g. participant privacy or use of data from a third party—those must be specified.

Reviewer #1: Yes

Reviewer #2: Yes

**********

4. Is the manuscript presented in an intelligible fashion and written in standard English?

PLOS ONE does not copyedit accepted manuscripts, so the language in submitted articles must be clear, correct, and unambiguous. Any typographical or grammatical errors should be corrected at revision, so please note any specific errors here.

Reviewer #1: Yes

Reviewer #2: Yes

**********

5. Review Comments to the Author

Please use the space provided to explain your answers to the questions above. You may also include additional comments for the author, including concerns about dual publication, research ethics, or publication ethics. (Please upload your review as an attachment if it exceeds 20,000 characters)

Reviewer #1: This is a technically sound and clearly documented investigation into the relaxivity of ferumoxytol and gadoterate at 0.25 T in phantoms accompanied by modelling to explain how the experimental results map onto a broader range of conditions.

The basis of the work is not very original, and the paper could be improved with more rigorous academic referencing of past work to position these results in context, but the results at 0.25 T seem to fill a small gap in the literature and will be of some benefit to an audience interested in applying similar contrast agents in this low magnetic field regime.

In terms of how the authors could generally improve their paper, I would recommend they expand their discussion of relaxation mechanisms at low field and how these relaxation effects are harnessed by particular imaging sequences. I would recommend the review article by Wahsner et al. (https://doi.org/10.1021/acs.chemrev.8b00363) as a good starting point for the broader context of selecting and applying contrast agents to specific environments.

I have few specific criticisms of the prose of the paper as a whole, which is largely written clearly and concisely, but I would like to mention a few specific issues that occurred to me:

1. On line 89 the authors say " However, an additional signal intensity (SI) gain because of higher relaxivities for the clinically interesting USPIO ferumoxytol [16] on a field strength lower than 1.5T has not been investigated." I find it a little unclear what the authors are trying to say here but I know for certain that USPIOs have been investigated at fields lower than 1.5T. Two investigations that spring to mind, and are particularly relevant to many of the points this paper makes are Yin et al. (https://doi.org/10.1038/s41598-018-30264-5) and Waddington et al. (DOI: 10.1126/sciadv.abb0998).

2. The paragraph beginning at line 217 seems unclear to me and I think would benefit from more explanation of how the bSFFP signal intensity varies with sequence parameters and the relative relaxivities of the samples. I think at several other points the rest of the paper would also benefit from this explanation, as that would help explain the banding artifacts and distortion seen in Fig 1.

3. The figures generally lack explanatory labels and helpful annotations to aid a reader and in the case of Fig1 there are also no colourbars. I appreciate that comparing across images may not always be a useful or fair comparison but I believe that it is crucial to still show the colour scales for each image so a reader can better interpret and evaluate the quality of the image.

Reviewer #2: This paper evaluates the effects on signal intensity due to a popular MRI contrast (agent gadoterate acid) and a promising alternative solution (ultra-small superparamagnetic iron-oxides) when images are taken in a low-field (0.25 T) MRI system. The research here undertaken is important to improve the performance and patient safety of low-field MRI systems when exploiting the benefits of contrast agents. The study compares simulations with phantom measurements for three MRI sequences often used with contrast agents. Results indicate that ultra-small superparamagnetic iron-oxide contrast agents can induce similar positive signal enhancement than as the gadolinium based counterpart, but with less contrast agent concentration, which can be beneficial for certain clinical imaging needs. The manuscript reads easily and the methods employed are appropriate.

Mayor issues:

p. 11, line 224 - From the SI vs CA plot of figures 3 and 4, it is possible to see that having the right concentration is crucial for USPIO because the SI gain is achieved over a narrower concentration range. Although simulations and measurements show qualitatively similar relaxivity curves, the CA concentration needed on measurements is 3 times larger than on simulations. Authors comment that this is likely due to an underestimation of T2 values. This argument would be solid if it would be estimated what is the T2 underestimation ratio that would justify the required concentration difference and if this is matches with what can be achieved by their MRI system.

p. 12, line 267- Authors conclude that ‘the use of ferumoxytol is more beneficial in generating positive contrast at low magnetic field strengths than gadoterate because lower concentrations yield equal signal enhancement’. This affirmation is misleading as it only considers a concentration advantage of USPIO, but does not account for other arguments in favor and against this contrast agent.

Minor issues:

p2, line 39- In the abstract it is not mentioned that, besides lower absolute SI increases, the measurements also show considerably lower relaxivities.

p3, line 67- Which characteristics 'stay equal'? Their effect on relaxation times?

p4, line 73- It would help the reader to briefly remind the reader why a reduction in T1 time generates positive contrast and a reduction in T2* generates negative contrast.

p4, line 85- What does MRA stand for?

p4, line 89- Typo ‘contrast agents types’

p7, line 137- Please, fix the missing reference.

p8, line 155- The table should go after its mention on the text, not before.

Discussion - It would enrich the discussion adding disadvantages of USPIO. For example, a very important application of CBCA is dynamic contrast enhanced (DCE) MRI. On this application the uptake curve is fitted to characterise certain tissue properties, and it is often required to run more than one CA dose. To this aim, a faster CA washout is beneficial. Showing USPIO a considerably slower washout time, it may be less convenient for such applications.

Discussion: It would be interesting to discuss whether the narrower concentration range at which the SI peaks has advantages and/or drawbacks. How important is the concentration on diagnosis with these contrast agents? Could some parts of the image have reduced SI enhancement due to too high/low concentration?

p10, line 215- Typo ‘as’ → ‘at’.

p11, line 237- Measurements may be missing the maximum of the peak on the USPIO phantom because sampling points are scarce.

p11, line 240- Please, rephrase.

p12, line 267- As mentioned before, using USPIO has benefits but can also have downsides (e.g, multi-dose DCE MRI). It may be convenient to rephrase the sentence not to mislead the reader. Also, the results do not show 'equal' signal enhancement as estated. The USPIO measurements reaches <%25 less signal enhancement than those of GD. See maximum values on figure 4.

Fig. 2- Although having a fixed colorbar range often helps comparing the different maps, in this case the range of the T2 maps is much smaller than that of the T1 maps. Visualisation of the T2 maps could benefit from adjusting the colorbar to a shorter range on both Gd and Fe T2 maps. Also, the text on these plots is unreadable due to image resolution. It may have been degraded by the submission system. Please, double check.

**********

6. PLOS authors have the option to publish the peer review history of their article (what does this mean?). If published, this will include your full peer review and any attached files.

If you choose “no”, your identity will remain anonymous but your review may still be made public.

**Do you want your identity to be public for this peer review?** For information about this choice, including consent withdrawal, please see our Privacy Policy.

Reviewer #1: No

Reviewer #2: **Yes: **Ruben Pellicer-Guridi

[NOTE: If reviewer comments were submitted as an attachment file, they will be attached to this email and accessible via the submission site. Please log into your account, locate the manuscript record, and check for the action link "View Attachments". If this link does not appear, there are no attachment files.]

While revising your submission, please upload your figure files to the Preflight Analysis and Conversion Engine (PACE) digital diagnostic tool, https://pacev2.apexcovantage.com/. PACE helps ensure that figures meet PLOS requirements. To use PACE, you must first register as a user. Registration is free. Then, login and navigate to the UPLOAD tab, where you will find detailed instructions on how to use the tool. If you encounter any issues or have any questions when using PACE, please email PLOS at figures@plos.org. Please note that Supporting Information files do not need this step.

# Author comment: Author response to Decision Letter 0

**DOI:** 10.1371/journal.pone.0256252.r002

2 Jul 2021

Answers on both reviewers comments were numbered and in the manuscript for each change it was indicated to which comment the change corresponds. With the text in bold our replies are stated below. 

Reviewer #1: 

This is a technically sound and clearly documented investigation into the relaxivity of ferumoxytol and gadoterate at 0.25 T in phantoms accompanied by modelling to explain how the experimental results map onto a broader range of conditions.

The basis of the work is not very original, and the paper could be improved with more rigorous academic referencing of past work to position these results in context, but the results at 0.25 T seem to fill a small gap in the literature and will be of some benefit to an audience interested in applying similar contrast agents in this low magnetic field regime.

Many thanks for your efforts in thoroughly reviewing this paper and your kind words. I have tried to answer your questions and remarks below and have adjusted the corresponding text in the manuscript. 

In terms of how the authors could generally improve their paper, I would recommend they expand their discussion of relaxation mechanisms at low field and how these relaxation effects are harnessed by particular imaging sequences. I would recommend the review article by Wahsner et al. (https://doi.org/10.1021/acs.chemrev.8b00363) as a good starting point for the broader context of selecting and applying contrast agents to specific environments.

0.1 Thanks for pointing out this interesting review. We expanded our discussion (as also indicated by the responses to specific criticisms below). Relaxation effects and their relation to MR imaging sequences were handled more extensively.

I have few specific criticisms of the prose of the paper as a whole, which is largely written clearly and concisely, but I would like to mention a few specific issues that occurred to me:

1. On line 89 the authors say " However, an additional signal intensity (SI) gain because of higher relaxivities for the clinically interesting USPIO ferumoxytol [16] on a field strength lower than 1.5T has not been investigated." I find it a little unclear what the authors are trying to say here but I know for certain that USPIOs have been investigated at fields lower than 1.5T. Two investigations that spring to mind, and are particularly relevant to many of the points this paper makes are Yin et al. (https://doi.org/10.1038/s41598-018-30264-5) and Waddington et al. (DOI: 10.1126/sciadv.abb0998).

1.1 We realize that there have been some investigations on lower field strengths and we should point this out. The proposed studies are performed at ultra-low field (<10mT), since in our study we make use of a 0.25T field strength, this is orders of magnitude different. However, those studies are very much relevant and are therefore mentioned now with respect to the current study. 

2. The paragraph beginning at line 217 seems unclear to me and I think would benefit from more explanation of how the bSFFP signal intensity varies with sequence parameters and the relative relaxivities of the samples. I think at several other points the rest of the paper would also benefit from this explanation, as that would help explain the banding artifacts and distortion seen in Fig 1.

1.2 The paragraph you mentioned seems indeed a bit unclear at first. Our reasoning why the bSSFP signal that we measured did not match with the simulations was that other factors like field inhomogeneities influence the signal more in bSSFP sequences. This also causes the banding that was sharply noticed as visible in Fig 1. We now elaborated more on why the experimental bSSFP signal was contradicting with the simulations. This explanation guides the reader further in understanding for what sequences USPIOs would be beneficial and what the drawbacks are of applying a bSSFP while using USPIOs.

3. The figures generally lack explanatory labels and helpful annotations to aid a reader and in the case of Fig1 there are also no colourbars. I appreciate that comparing across images may not always be a useful or fair comparison but I believe that it is crucial to still show the colour scales for each image so a reader can better interpret and evaluate the quality of the image.

1.3 Because all images were equally scaled and the units are arbitrary we first decided to not include a colorbar. However, to make clear that this is the case, we agree interpretation of the images would indeed benefit from this and we added the colorbar. Furthermore, Fig 2 and its caption were revised with removal of abundant information and (white) space. This was also in line with the comments of reviewer #2 on the lack of a colorbar and lower image quality. Fig 3 and Fig 4 were exported in higher quality and their captions were supplemented where necessary. Besides, the width of Fig 4 is now more compressed in order to compare better with the results in Fig 3. 

Reviewer #2: This paper evaluates the effects on signal intensity due to a popular MRI contrast (agent gadoterate acid) and a promising alternative solution (ultra-small superparamagnetic iron-oxides) when images are taken in a low-field (0.25 T) MRI system. The research here undertaken is important to improve the performance and patient safety of low-field MRI systems when exploiting the benefits of contrast agents. The study compares simulations with phantom measurements for three MRI sequences often used with contrast agents. Results indicate that ultra-small superparamagnetic iron-oxide contrast agents can induce similar positive signal enhancement than as the gadolinium based counterpart, but with less contrast agent concentration, which can be beneficial for certain clinical imaging needs. The manuscript reads easily and the methods employed are appropriate.

Many thanks for your kind words and efforts in thoroughly reading our manuscript. The issues that you raised were well considered and below we explained and discussed how we improved our manuscript based on your advice.

Mayor issues:

p. 11, line 224 - From the SI vs CA plot of figures 3 and 4, it is possible to see that having the right concentration is crucial for USPIO because the SI gain is achieved over a narrower concentration range. Although simulations and measurements show qualitatively similar relaxivity curves, the CA concentration needed on measurements is 3 times larger than on simulations. Authors comment that this is likely due to an underestimation of T2 values. This argument would be solid if it would be estimated what is the T2 underestimation ratio that would justify the required concentration difference and if this is matches with what can be achieved by their MRI system.

2.1 This is an excellent issue that you are pointing out. First of all, the width of Fig 3 and Fig 4 was not matching, making it harder to interpret and compare the maximum SI as function of concentration between experiments and simulations. Where Fig 4 has only three plots with CA ranging from 0-15 mM, Fig 3 has six plots and thus more compressed graphs. We fixed Fig 4 so it matched the range in Fig 3 better.

The text regarding the underestimation of T2 values in the discussion (original starting on line 224) was also adjusted based on the comments of Reviewer #1. We elaborated on why this underestimation occurred and what it meant for our data. Since magnetic field imperfections led to lower signal values in the bSSFP sequence that were used for calculation of the T2 values, the estimated r2 relaxivity was probably much higher than the true value. We explained in this paragraph that the actual r2 of ferumoxytol and gadoterate are more likely between 80-120 and 3-5 mM-1 s-1 respectively. Simulations using these extrapolated literature values does result in curves that are much more like the experimental curves. The exact T2 values that we found for ferumoxytol and that were used in the fit are 123, 23, and 11 ms. T2 values of 123, 52, and 26 ms would result in an expected relaxivity of 100 mM-1 s-1. For such short T2 values a small difference causes rapid offset in the r2 fit. However, the signal we measured was more T2* dependent and thus smaller than T2, as we explained in the discussion. This offset seemed logical in our view, but based on your issue we made our explanation regarding this much more clear.

p. 12, line 267- Authors conclude that ‘the use of ferumoxytol is more beneficial in generating positive contrast at low magnetic field strengths than gadoterate because lower concentrations yield equal signal enhancement’. This affirmation is misleading as it only considers a concentration advantage of USPIO, but does not account for other arguments in favor and against this contrast agent.

2.2 Our conclusion was indeed somewhat misleading as we considered the advantage based on relaxivity characteristics alone. We made this more specific in our conclusion.

Minor issues:

p2, line 39- In the abstract it is not mentioned that, besides lower absolute SI increases, the measurements also show considerably lower relaxivities.

2.3 You are correct in pointing out that the results section in our abstract did not fully match the results from the manuscript. The estimated r2 relaxivity was indeed very high (probably due to imperfections in our system), which was reflected in the simulations. However, in the final measurements the signal of course depends on the real relaxivity of the CAs. These lower relaxivities in the experiments are causing a mismatch, which was mainly visible in the bSSFP graphs. We corrected this sentence in the abstract.

p3, line 67- Which characteristics 'stay equal'? Their effect on relaxation times? 

2.4 Yes their effect on relaxation times. We made this more specific in the manuscript.

p4, line 73- It would help the reader to briefly remind the reader why a reduction in T1 time generates positive contrast and a reduction in T2* generates negative contrast.

2.5 This is a very nice point to address and greatly helps our reader in understanding the study. We added some sentences on that location to make this more clear.

p4, line 85- What does MRA stand for?

2.6 Magnetic resonance angiography. We removed the acronym since it was only used once.

p4, line 89- Typo ‘contrast agents types’

2.7 Fixed.

p7, line 137- Please, fix the missing reference.

2.8 Fixed. (was due to converting by the upload system)

p8, line 155- The table should go after its mention on the text, not before.

2.9 The missing reference was also Table 2, so this mention is now correct.

Discussion - It would enrich the discussion adding disadvantages of USPIO. For example, a very important application of CBCA is dynamic contrast enhanced (DCE) MRI. On this application the uptake curve is fitted to characterise certain tissue properties, and it is often required to run more than one CA dose. To this aim, a faster CA washout is beneficial. Showing USPIO a considerably slower washout time, it may be less convenient for such applications.

2.10 We agree this point is valuable to add to the discussion, so we added a paragraph where this relevant drawback of the longer retention time was mentioned. 

Discussion: It would be interesting to discuss whether the narrower concentration range at which the SI peaks has advantages and/or drawbacks. How important is the concentration on diagnosis with these contrast agents? Could some parts of the image have reduced SI enhancement due to too high/low concentration?

2.11 There are indeed some more drawbacks and advantages related to using the right concentration in clinical practice. For example, a narrow peak means that the concentration should be in that range and in that range only to give positive contrast. Although this seems hard to achieve, the blood-pool residence time of ferumoxytol is much longer compared to GBCAs, making this easier to achieve. Subsequent doses (at least on the same day of imaging) are also not necessary. After administration of well-known doses, ferumoxytol spreads evenly in the blood-pool. We also referred now to the mini-review of Vasanawala et al. (2016, MRM), which elaborates further on safety and techniques of ferumoxytol administration.

p10, line 215- Typo ‘as’ → ‘at’.

2.12 We apologize for the confusion. We meant to say that a low concentration of ferumoxytol leads to similar signal enhancement as a much higher concentrations of gadoterate. Nevertheless, this sentence has been made a bit shorter and hopefully more clear.

p11, line 237- Measurements may be missing the maximum of the peak on the USPIO phantom because sampling points are scarce.

2.13 That is correct, and we clarified this in the discussion. 

p11, line 240- Please, rephrase.

2.14 Rephrased the sentence.

p12, line 267- As mentioned before, using USPIO has benefits but can also have downsides (e.g, multi-dose DCE MRI). It may be convenient to rephrase the sentence not to mislead the reader. Also, the results do not show 'equal' signal enhancement as estated. The USPIO measurements reaches <%25 less signal enhancement than those of GD. See maximum values on figure 4.

2.15 We edited the text in the conclusion to match this comment and the major issue that was mentioned earlier.

Fig. 2- Although having a fixed colorbar range often helps comparing the different maps, in this case the range of the T2 maps is much smaller than that of the T1 maps. Visualisation of the T2 maps could benefit from adjusting the colorbar to a shorter range on both Gd and Fe T2 maps. Also, the text on these plots is unreadable due to image resolution. It may have been degraded by the submission system. Please, double check.

2.16 Fig 2 was extended with a color bar with ranges corresponding to the values that were found in both maps. Also, the caption was clarified which was in line with the remarks of Reviewer #1. All figures were double checked on export in highest possible quality.

________________________________________

Because of the thorough review of our manuscript we came across the following paragraph (original starting at p. 11 line 243) in our discussion that has become obsolete. This information was based on previous experiments that were conducted for this manuscript in a demi-water solvent. However, with bovine blood the absolute difference between simulations and experiments was less pronounced. We apologize for this insufficiency, and have removed those sentences.

Attachment Submitted filename: Response to Reviewers.docx Click here for additional data file.

# Peer review: Decision Letter 1

**DOI:** 10.1371/journal.pone.0256252.r003

**Authors:** Viktor Vegh

4 Aug 2021

Comparing the Signal Enhancement of a Gadolinium Based and an Iron-Oxide Based Contrast Agent in Low-Field MRI

PONE-D-21-15569R1

Dear Dr. van Zandwijk,

We’re pleased to inform you that your manuscript has been judged scientifically suitable for publication and will be formally accepted for publication once it meets all outstanding technical requirements.

Within one week, you’ll receive an e-mail detailing the required amendments. When these have been addressed, you’ll receive a formal acceptance letter and your manuscript will be scheduled for publication.

An invoice for payment will follow shortly after the formal acceptance. To ensure an efficient process, please log into Editorial Manager at http://www.editorialmanager.com/pone/, click the 'Update My Information' link at the top of the page, and double check that your user information is up-to-date. If you have any billing related questions, please contact our Author Billing department directly at authorbilling@plos.org.

If your institution or institutions have a press office, please notify them about your upcoming paper to help maximize its impact. If they’ll be preparing press materials, please inform our press team as soon as possible -- no later than 48 hours after receiving the formal acceptance. Your manuscript will remain under strict press embargo until 2 pm Eastern Time on the date of publication. For more information, please contact onepress@plos.org.

Kind regards,

Viktor Vegh, Ph.D.

Academic Editor

PLOS ONE

Additional Editor Comments (optional):

Reviewers' comments:

Reviewer's Responses to Questions

**Comments to the Author**

1. If the authors have adequately addressed your comments raised in a previous round of review and you feel that this manuscript is now acceptable for publication, you may indicate that here to bypass the “Comments to the Author” section, enter your conflict of interest statement in the “Confidential to Editor” section, and submit your "Accept" recommendation.

Reviewer #1: All comments have been addressed

Reviewer #2: All comments have been addressed

**********

2. Is the manuscript technically sound, and do the data support the conclusions?

The manuscript must describe a technically sound piece of scientific research with data that supports the conclusions. Experiments must have been conducted rigorously, with appropriate controls, replication, and sample sizes. The conclusions must be drawn appropriately based on the data presented. 

Reviewer #1: Yes

Reviewer #2: Yes

**********

3. Has the statistical analysis been performed appropriately and rigorously? 

Reviewer #1: Yes

Reviewer #2: N/A

**********

4. Have the authors made all data underlying the findings in their manuscript fully available?

The PLOS Data policy requires authors to make all data underlying the findings described in their manuscript fully available without restriction, with rare exception (please refer to the Data Availability Statement in the manuscript PDF file). The data should be provided as part of the manuscript or its supporting information, or deposited to a public repository. For example, in addition to summary statistics, the data points behind means, medians and variance measures should be available. If there are restrictions on publicly sharing data—e.g. participant privacy or use of data from a third party—those must be specified.

Reviewer #1: Yes

Reviewer #2: (No Response)

**********

5. Is the manuscript presented in an intelligible fashion and written in standard English?

PLOS ONE does not copyedit accepted manuscripts, so the language in submitted articles must be clear, correct, and unambiguous. Any typographical or grammatical errors should be corrected at revision, so please note any specific errors here.

Reviewer #1: Yes

Reviewer #2: Yes

**********

6. Review Comments to the Author

Please use the space provided to explain your answers to the questions above. You may also include additional comments for the author, including concerns about dual publication, research ethics, or publication ethics. (Please upload your review as an attachment if it exceeds 20,000 characters)

Reviewer #1: (No Response)

Reviewer #2: (No Response)

**********

7. PLOS authors have the option to publish the peer review history of their article (what does this mean?). If published, this will include your full peer review and any attached files.

If you choose “no”, your identity will remain anonymous but your review may still be made public.

**Do you want your identity to be public for this peer review?** For information about this choice, including consent withdrawal, please see our Privacy Policy.

Reviewer #1: No

Reviewer #2: **Yes: **Ruben Pellicer-Guridi

# Editor report: Acceptance letter

**DOI:** 10.1371/journal.pone.0256252.r004

**Authors:** Viktor Vegh

9 Aug 2021

PONE-D-21-15569R1 

Comparing the Signal Enhancement of a Gadolinium Based and an Iron-Oxide Based Contrast Agent in Low-Field MRI 

Dear Dr. van Zandwijk:

I'm pleased to inform you that your manuscript has been deemed suitable for publication in PLOS ONE. Congratulations! Your manuscript is now with our production department. 

If your institution or institutions have a press office, please let them know about your upcoming paper now to help maximize its impact. If they'll be preparing press materials, please inform our press team within the next 48 hours. Your manuscript will remain under strict press embargo until 2 pm Eastern Time on the date of publication. For more information please contact onepress@plos.org.

If we can help with anything else, please email us at plosone@plos.org. 

Thank you for submitting your work to PLOS ONE and supporting open access. 

Kind regards, 

PLOS ONE Editorial Office Staff

on behalf of

Dr. Viktor Vegh 

Academic Editor

PLOS ONE
